# The effects of killer cell immunoglobulin-like receptor (KIR) genes on susceptibility to severe COVID-19 in the Iranian population

**DOI:** 10.1186/s12865-024-00631-1

**Published:** 2024-06-28

**Authors:** Narges Karami, Shaghik Barani, Mona Fani, Seppo Meri, Reza Shafiei, Kurosh Kalantar

**Affiliations:** 1 Department of Immunology, School of Medicine, Shiraz University of Medical Sciences, Shiraz, 71348-45794 Iran; 2 Infection and Immunity Program and Department of Biochemistry and Molecular Biology, Biomedicine Discovery Institute, Monash University, Clayton, VIC Australia; 3 Vector-borne Diseases Research Center, North Khorasan University of Medical Sciences, Bojnurd, Iran; 4 Department of Bacteriology & Immunology and Translational immunology Research Program, University of Helsinki Diagnostic Center, Helsinki University Central Hospital, University of Helsinki, Helsinki, Finland; 5 Autoimmune Diseases Research Center, Shiraz University of Medical Sciences, Shiraz, Iran

**Keywords:** COVID-19, Killer cell immunoglobulin-like receptors (KIRs), NK cell

## Abstract

**Background:**

Variations in the innate and adaptive immune response systems are linked to variations in the severity of COVID-19. Natural killer cell (NK) function is regulated by sophisticated receptor system including Killer-cell immunoglobulin-like receptor (*KIR*) family. We aimed to investigate the impact of possessing certain *KIR* genes and *genotypes* on COVID19 severity in Iranians. KIR genotyping was performed on 394 age/sex matched Iranians with no underlying conditions who developed mild and severe COVID- 19. The presence and/or absence of 11 *KIR* genes were determined using the PCR with sequence specific primers (PCR-SSP).

**Results:**

Patients with mild symptoms had higher frequency of*KIR2DS1* (*p* = 0.004) and *KIR2DS2* (*p* = 0.017) genes compared to those with severe disease. While *KIR3DL3* and deleted variant of KIR2DS4 occurred more frequently in patients who developed a severe form of the disease. In this study, a significant increase of and B haplotype was observed in the Mild group compared to the Severe group (respectively, *p* = 0.002 and *p* = 0.02). Also, the prevalence of haplotype A was significantly higher in the Severe group than in the Mild group (*p* = 0.02).

**Conclusions:**

These results suggest that the KIR2DS1, KIR2DS, and B haplotype maybe have a protective effect against COVID-19 severity. The results also suggest the inhibitory gene *KIR2DL3* and haplotype A are risk factors for the severity of COVID-19.

**Supplementary Information:**

The online version contains supplementary material available at 10.1186/s12865-024-00631-1.

## Background

COVID-19 is a severe acute respiratory syndrome caused by a highly contagious virus known as severe acute respiratory syndrome coronavirus 2 (SARS-CoV-2) [1]. The COVID-19 outbreak led to extensive global morbidity and mortality [2]. According to the report of the World Health Organization (WHO) released on August 2023, around 7 million deaths by COVID-19 have been recorded (https://covid19.who.int/table) [3]. The symptoms of COVID-19 can vary from asymptomatic infection which partially remains unrecognized to mild and severe disease with fever, cough, shortness of breath, pneumonia and organ failure [4–7]. Natural Killer (NK) cells play a crucial role in innate immune response as the first line of defense against infection with SARS-CoV-2 [8, 9]. NK cells are equipped with a diverse array of inhibitory and activating receptors including killer cell immunoglobulin-like receptors (KIRs) which have been involved in immune responses to viral infection and tumors [10]. Interaction of inhibitory and activating KIRs with HLA class I molecules on target cells determines the responsiveness or tolerance of NK cells [11]. As stated in “Missing self” hypothesis, NK cells check and differentiate healthy cells from unhealthy targets such as virally infected or transformed cells by recognizing down-regulation of human leukocyte antigen-I (HLA-I) or overexpression of activating ligands on their cell surface [12]. *KIR* gene family encode receptors with two or three extracellular domains (D) involved in antigen recognition and short or long cytoplasmic tail providing activating and inhibitory signals respectively [13]. The gene content in Killer Cell Immunoglobulin-Like Receptors (KIRs) in Natural Killer (NK) cells includes 11 expressed human *KIR* genes. Among these genes, *KIR2DS1*, 2, 3, 4, and 5 are activating receptors, while *KIR2DL1*, *2DL2/3*, and *2DL5* are inhibitory receptors. *KIR2DL4* has the potential for both activating and inhibitory functions, and KIR3DL1/S1 has mutually exclusive subsets of allotypes with activating (*KIR3DS1*) and inhibitory properties [14]. This diversity in gene content contributes to the functional diversity of *KIR*s and their role in modulating NK cell functions through interactions with their ligands, particularly HLA class I molecules. Various combinations of *KIR* genes are inherited as A and B haplotypes consisting of *KIRDL3*, *KIR3DP1 KIR2DL4*, and *KIR3DL2* as framework genes present in all *KIR* haplotypes along with each haplotype associated activating and inhibitory genes [15]. KIR haplotypes are divided into centromeric half by *KIRDL3* and *KIR3DP1* and telomeric region by *KIR2DL4* and *KIR3DL2.* [16, 17].

Recent studies have suggested that carrying certain KIRs were associated with developing a severe form of COVID-19 [18–20]. These genetic variations can affect the activity and function of NK cells to recognize and eliminate infected cells. This gene variant has been found to enhance the activation of NK cells, leading to an exaggerated immune response and the development of severe symptoms [6, 20, 21]. We aimed to define the association between certain KIR genotypes with severity of disease in COVID-19 in an Iranian population.

## Materials and methods

### Study population

This study involved 394 patients with COVID-19 who were admitted to Faghihi Hospital of Shiraz University of Medical Sciences between June 1 and December 1, 2021.Real-time polymerase chain reaction (RT-PCR) on nasopharyngeal swabs was used to confirm the diagnosis of SARS-CoV-2 infection. Participants were classified into two groups of hospitalized patients (*n* = 198) with severe disease who received high-flow nasal oxygen supplementation or ventilation, and mild disease group (*n* = 196) having mild upper respiratory tract infection including loss of smell or taste runny nose, headache, dry cough and flu-like symptoms.

The sample size calculation indicated that a minimum of 195 patients is necessary to maintain a precision of 2% in the 95% confidence interval for estimating the prevalence of genotypes in the studied population.

### Sampling, DNA extraction and KIRs genotyping

Peripheral blood samples (2mL) were collected from COVID-19 patients. Samples were collected according to the rules and regulations of the Human Research Ethics Committee of Shiraz University of Medical Sciences (IR.SUMS.REC.1400.462). Informed consent was obtained from participants prior to participating in this research.

DNA was extracted using the salting-out method [22]. In brief, 2 mL of whole blood was added to 1 mL lysis buffer (0.15 M NaCl, 5mM EDTA pH 8.0 and 2% (w/v) SDS, and proteinase K. The mixture was incubated at 55–65 °C overnight to digest proteins and cellular debris. Then a high concentration of saturated NaCl was added to the mixture, which decreases protein solubility due to the high salt concentration, resulting in protein precipitation. The mixture was then centrifuged at 2500 rpm for 15 min, and the DNA-containing supernatant was pipetted into a fresh tube. The DNA can then be precipitated using ethanol (70%), and the precipitate was washed with ethanol to remove remaining contaminants(75%). The precipitated DNA was then dissolved in an aqueous buffer like Tris-EDTA or nuclease-free water and is ready for use in downstream applications.

KIR genotyping of 11 KIR genes (*KIR2DL1*, *KIR2DL2*, *KIR2DL3*, *KIR3DL1*, *KIR2DL5*, *KIR2DS1*, *KIR2DS2*, *KIR2DS3*, *KIR2DS5*, *KIR3DS1*, and *KIR2DS4*) was completed by sequence specific primers-polymerase chain reaction (PCR-SSP) method, as described in previous studies [15, 23, 24]. In brief, in the case *KIR* genotyping using PCR-SSP, the process involves the use of a set of primers that are specific to each *KIR* gene of interest (Supplementary Table 1). These primers are designed to bind to specific regions within the *KIR* genes, allowing for the amplification of those regions when the PCR reaction is performed. By comparing the amplified products to a reference, the absence or presence of each *KIR* gene can be determined.

For adjusting the protocol, 100 ng of genomic DNA was subjected to PCR using 5 µl of Taq DNA Polymerase 2x Master Mix (Parstous, iran). PCR was conducted in the following circumstances: “Two minutes of initial denaturation at 95°C, followed by 10 cycles of 10 s at 94°C and 40 s and 65°C respectively; and then 20 cycles of 94°C for 20 s, 61°C for 20 s and 72°C for 30 s”.

Around 5 µl of the PCR product was stained with dye safe stain (Pars Tous, Iran) and then load in 2% agarose gel and in 4% agarose gel for segregation of the KIR2DS4 full and deleted variants. The gel was viewed with a GelDoc (BIO-RAD, U.S.A).

PCR product size for each *KIR* gene was detected by comparing the band size against DNA ladder (Pars Tous, Iran). *KIR* *genotypes* (Bx, AA), A and B haplogroups, clusters and Bx subsets were defined according to individuals *KIRs* content using specific formulas described previously [15].

### Statistical analysis

Data were analyzed using SPSS statistical software (version 20). The frequency of each gene and haplotype was compared between mild and severe groups. The *p*-value < 0.05 was considered statistically significant for two-sided Chi-square test with Yates’s correction to adjust the differences. The odds ratio (OR) and 95% confidence interval (CI) were calculated in order to examine the magnitude and statistical significance of the association.

## Results

### Demographic characteristics

In this study, 394 patients with COVID-19 were divided into two groups matched for age and sex. Patients were from Fars province which is located in the south of Iran. The mean age for patients in severe disease group was 40.4 ± 9.2 and 40.5 ± 9.8 for patients with mild illness (Table 1).

**Table 1:** Demographic characteristics of COVID-19 patients

Group	*N* (M vs. F)	Mean age	Std Deviation	Range
severe cases	198 (119vs79)	40.4	9.2	9.1–63
mild cases	196 (118 vs. 78)	40.5	9.7	9.7–64
Total	394(237 − 157)	40.4	9.4	9.4–64

M = Male, F = Female

**Fig. 1:**
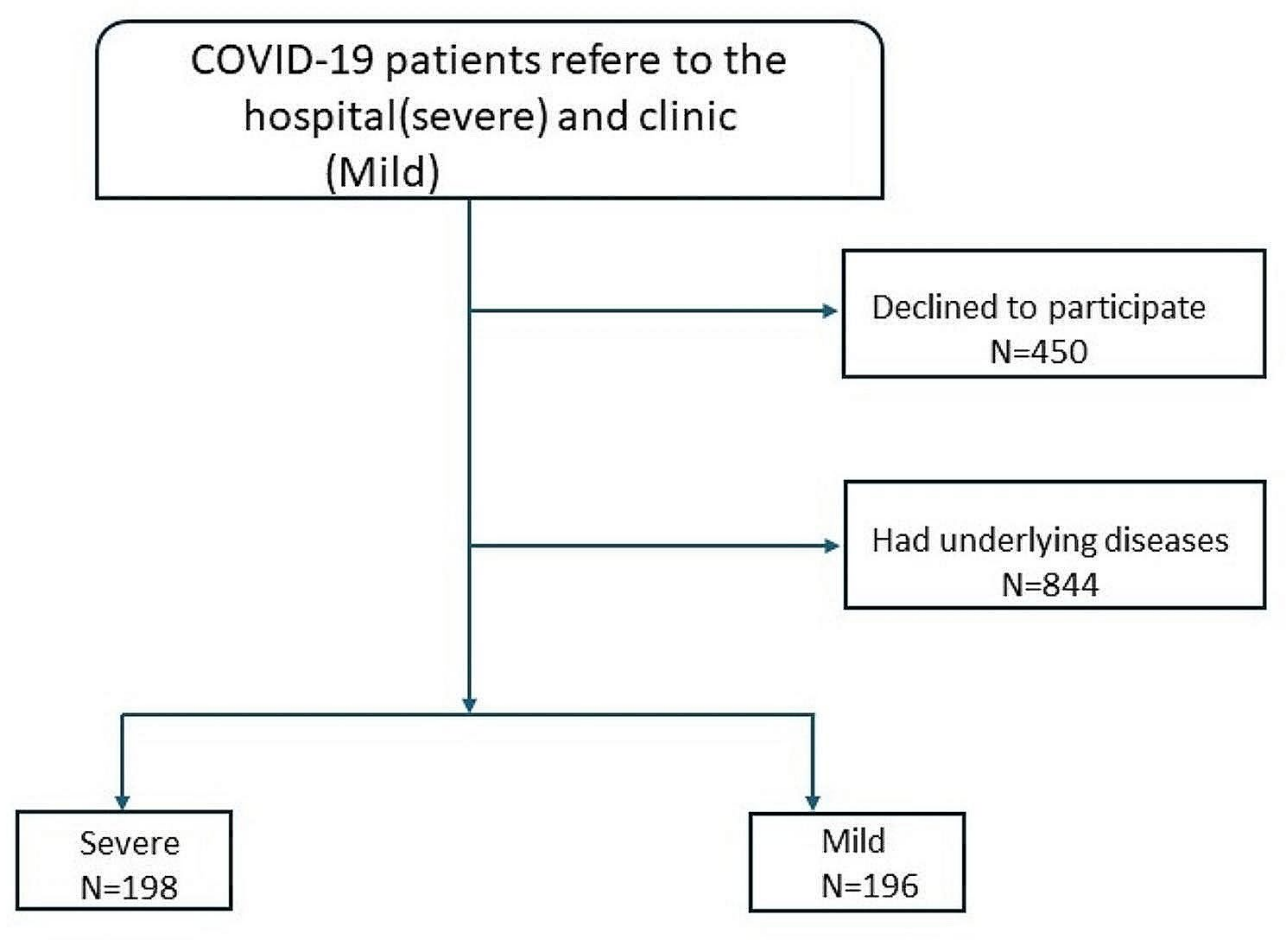
Flow diagram of Inclusion/exclusion of patients

This study excluded patients with common underlying diseases including diabetes, hypertension, cardiovascular disease, autoimmunity and cancer (Fig. 1).

### Predisposing role of KIR2DS1 and KIR2DS2 and protective role of KIR2DL3 and deleted variant of KIR2DS4 in development of severe form of COVID-19

To examine the potential contribution of *KIR* genes and genotypes as a genetic risk factor for COVID-19 severity, the *KIR* genes frequency was compared in to groups classified as mild and severe (Table 2).

**Table 2:** Comparing the carrier frequency of KIR genes in the groups with severe or mild disease

KIR gene		severe disease *N* = 198	mild disease *n* = 196	severe vs. mild disease
		*N* %F	*N* %F	*p*-value OR (95% CI)
A haplotype associated genes	*2DL1*	195 98.5%	194 99.0%	NS
	*2DL3*	183 92.4%	160 81.6%	0.001* **1.132** **1.048–1.223**
	*3DL1*	192 97%	182 92.9%	NS
	*2DS4*	189 95.5%	186 94.9%	NS
	*2DS4 fl.*	7 3.5%	12 6.1%	NS
	*2DS4 del*	144 72.7%	123 62.8%	0.03* **1.58** **1.032–2.429**
	*2DS4 fl/del*	38 19.2%	51 26%	NS
B haplotype associated genes	*2DS1*	65 32.8%	92 46.9%	0.004* **0.553** **0.366–0.831**
	*2DS2*	107 54%	129 65.8%	0.017* **0.611** **0.406–0.917**
	*2DS3*	85 42.9%	97 49.5%	NS
	*2DS5*	48 24.2%	54 27.6%	NS
	*3DS1*	77 38.9%	59 30.1%	NS
	*2DL2*	131 66.2%	120 61.2%	NS
	*2DL5*	106 53.5%	119 60.7%	NS

2DS4del: KIR2DS4 deleted variant, 2DS4fl: KIR2DS4 full length, 2DS4fl, del: both KIR2DS4 deleted variant and full length.; * *p* < 0.05: statistically significant; based on two-tailed Chi-square with Yates’ correction; NS = non-significant

A signifiant decrease was found in the carrier frequency of activating *KIR2DS1* (*p* = 0.004, OR = 0.553, CI = 0.366–0.831) and *KIR2DS2* (*p* = 0.017, OR = 0.611, CI = 0.406–0.917) in patients with severe symptoms compared to patients with mild disease. In case of inhibitory *KIRs*, patients carrying *KIR2DL3* constituted a higher proportion in severe group when compared with the mild one (*p* = 0.001, OR = 1.132, CI = 1.048–1.223).

KIR2DS4 variants distribution were analyzed by classifying patients into three groups as *2DS4fl, 2DS4del* and 2DS4 fl., del according to the presence/absence of full and deleted variants both in tandem and apart [13]. The COVID-19 patients with severe disease had a greater prevalence of deleted variant of *KIR2DS4* compared to the mild group (*p* = 0.03, CI = 1.032–2.429 ، OR = 1.58). These finding may indicate that activating *KIR2DS1* and *KIR2DS2* confer protection against developing severe disease while *KIR2DS4del* and inhibitory *KIR2DL3* increase the risk of severe disease in patients with COVID-19.

### The protective role of BB genotype, B haplotype, and the association of a haplotype with the severity of COVID-19

Based on the presence and absence of activating and inhibitory KIRs, A and B haplotypes were determined for each individual. Genotype consists of KIR A haplotype associated genes *3DL3*, *2DL1*, *2DL3*, *2DL4*, *3DL1*, *2DS4* and *3DL2* were referenced to homozygous group A haplotype or KIR AA genotype. The remaining *genotypes* possessing at least one or more B haplotype associated genes (*2DS1*, *2DS2*, *2DS3*, *2DS5*, *3DS2* and *2DL5*) and thus having one (heterozygous for A and B haplotypes) or two B haplotypes (homozygous for B haplotypes) were defined as KIR Bx genotype [10].

The B haplotype was observed with a higher frequency in the mild disease group than in the severe disease group was observed (*p* = 0.02, OR = 0.714, CI = 0.539–0.945). The A haplotype was observed more frequently in the severe disease group (*p* = 0.02, OR = 1.4, CI = 1.058–1.855). According to the linkage disequilibrium between centromeric and telomeric haplotypes, two distinct *KIR* genes clusters are defined as C4 (*KIR2DS2*-*2DL2*-*2DS3*-*2DL5B*) and T4 (*KIR3DS1*-*2DL5A*-*2DS5*-*2DS1* clusters which frequently occurred in individuals [25].

Based on the presence of four genes or the absence of one or more genes in each cluster, the Bx *genotype* is divided into 4 subgroups: including C4Tx, CxT4, C4T4, and CxTx (Table 3). The picture of haplotype of studied group have considered in a supplementary Table 2.

**Table 3:** The frequency of the KIR genotypes and haplotypes in the study population

KIR	severe disease *n* = 198	mild disease *n* = 196	severe vs. mild
	*N* %F	*N* %F	*p*-value OR (95% CI)
AA genotype	34 16.7%	25 12.8%	NS
Bx genotype	164 82.8%	171 87.2%	NS
Bx genotype	*N* = 164	*N* = 171	
C4Tx genotype	38 23.17%	54 31.57%	NS
CxT4 genotype	10 6%	10 5.8%	NS
C4T4 genotype	6 3.6%	7 4.09%	NS
CxTx genotype	110 68.12%	100 58.4%	NS
A haplotype	208 52.5%	173 44.1%	0.02* **1.4** **1.058–1.855**
B haplotype	188 47.4	219 55.8%	0.02* **0.714** **0.539–0.945**
C4 gene cluster	45 27%	63 36.4%	NS
T4 gene cluster	16 9.6%	18 13.8%	NS

The frequencies of the haplotypes A and B were determined by the following formulas [26]: Haplotype A: 2nAA + nAB/2 N, Haplotype B: 2nBB + nAB/2 N. (nAA: number of AA genotype, nAB: number of AB genotype, nBB: number of BB genotype). *p < 0.05: statistically significant; based on two-tailed Chi square with Yates’ correction [25]. NS = non-significant

### Association of the number of inherited inhibitory KIR (iKIR) and activating KIR (aKIR) genes with the protection or severity of COVID-19

*KIR* genes are inherited in different combinations and various number of activating and inhibitory genes. Carriers of Bx *genotypes* can express 2 to 6 activating KIRs and up to six inhibitory KIRs whereas individuals with AA genotype may represent one activating *KIR2DS4* and four inhibitory *KIR*s.

As displayed in Table 4, individuals with greater number of activating than inhibitory KIRs (aKIRs > iKIRs) constituted a higher proportion of COVID-19 patients with mild symptoms than severe disease (*p* = 0.01, OR = 0.530, CI = 0.305–0.909) while patients with severe COVID-19 carried more inhibitory than activating KIRs (iKIRs > aKIRs: *p* = 0.01, OR = 1.7, CI = 1.11–2.263). In addition, In our study, individuals in the severe disease group mostly inherited only one activating gene (*p* = 0.014, OR = 1.650, CI = 1.098–2.479), while most individuals in the mild disease group had 3 activating genes (*p* = 0.031, OR = 0.683, CI = 0481 − 0.970).

.

**Table 4:** Distribution of different KIR numbers and comparisons of KIR numbers between the severe and mild disease groups

KIR genes number	Severe disease *n* = 198	Mild disease *n* = 196	Severe vs. mild disease
	*N* %F	*N* %F	*p*-value OR (95% CI)
*iKIR = 1*	0 0%	0 0%	NS
*iKIR = 2*	2 1%	4 2%	NS
*iKIR = 3*	50 25.3%	62 31.6%	NS
*iKIR = 4*	80 40.4%	70 35.7%	NS
*iKIR = 5*	66 33.3%	60 30.63%	NS
*aKIR = 1*	50 25.3%	30 15.3%	0.014* **1.650** **1.098–2.479**
*aKIR = 2*	39 19.7%	31 15.8%	NS
*aKIR = 3*	40 20.2%	58 29.6%	0.031* **0.683** **0.481–0.970**
*aKIR = 4*	33 16.7%	42 21.4%	NS
*aKIR = 5*	31 15.7%	28 14.3%	NS
*aKIR = 6*	4 2%	7 3.6%	NS
*aKIRs > iKIRs*	25 12.6%	42 21.4%	0.01* **0.530** **0.305–0.909**
*iKIRs = aKIRs*	26 13.1%	31 15.8%	**NS**
*iKIRs > aKIRs*	147 74.2%	123 62.8%	0.01* **1.7** **1.11–2.263**

*P < 0.05; two-tailed Chi-square with Yates’ correction

## Discussion

The majority of patients with COVID-19 are asymptomatic or have only mild symptoms. However, some patients suffer from a severe or critical form of the disease that is complicated [27]. The clinical manifestations of COVID-19 are influenced by the host immune system, viral load, environmental factors and various types of co-morbidities, especially obesity and previous respiratory or cardiac illnesses [28].

The innate immune cells, especially NK cells, belong to the first line of defense against viruses [29]. Unlike T lymphocytes, their target identification is not specific. NK cell activation depends on the baance between activating and inhibitory KIRs and their ligands [30]. One interesting finding in this study is an association between some KIR genotypes and COVID-19 disease severity. The frequency of *KIR2DS1* and *KIR2DS2* genes in the mild disease subjects was higher than in the severely ill patients. In support of this, a study performed in Italy showed that the *KIR2DS2* gene frequency was significantly increased in the mild form of COVID-19 compared to the severe one. In addition, their findings showed that the combination of *KIR2DS2/HLA-C1* was significantly more common in asymptomatic or in patients with mild disease [6]. Another investigation also emphasized a protective role for activating KIRs in hospitalized COVID-19 patients admitted to the ICU. The KIR2DS5 gene was significantly associated with the recovery from a severe form of COVID-19 [31]. In addition to COVID-19, the protective role of *KIR2DS2* has been reported in other viral diseases. For example, the activator gene *KIR2DS2* has a protective role against *Cytomegalovirus* infection in kidney transplantation [32]. Bonagura et al. reported a possible role of activating receptors, such as KIR3DS1 and KIR2DS1, in generating an effective primary immune response against HPV to induce resistance to HPV-induced recurrent respiratory papillomatosis [33]. NK cell activity relies on a balance of inhibitory and activating receptor signals to determine which target cells are attacked or tolerated.

A comparison of the findings with other studies confirms that the balance of KIRs signals in the mild disease group was in favor of NK cell activation, resulting in early virus clearance and prevention of severe clinical manifestations. Contrary to the findings of COVID-19, the *KIR3DS1*, *KIR2DS5*, and KIR2DL5 genes are associated with the severity of influenza A disease [34]. The researchers in this study suggested that high numbers of KIR-activating genes may led to stronger NK cell activation and severe inflammation. However, the small sample size and non-exclusion of patients with underlying diseases as diabetes, which can affect susceptibility to severe types of influenza, were the limitations of this study. Regarding the inhibitory receptors, our finding indicates that the frequency of the inhibitory gene KIR2DL3 was significantly higher in the severe disease group. In addition, the frequency of the inhibitory genes 3DL1 and 2DL2 was higher in the severe disease group than in the mild group. The results of Meira Leite et al. showed that the inhibitory gene KIR2DL3 and the AA genotype were associated with the daily mortality rate in patients, while there was a negative association between the activating gene *KIR2DS2* and the daily mortality rate [35]. Furthermore, the study by Hajeer et al. also suggested that KIR3DL1 genes are related to an increased risk for severe COVID-19 [20]. The general conclusion of these studies is thus that inhibitory KIR genes are associated with the severe form of COVID-19.

Inhibitory KIRs reduce the antiviral response of NK cells, allowing an increased viral replication. This could lead to the recall of other immune cells to the lungs, cytokine storm and ARDS in the severe form of COVID-19. In another study, researchers showed that KIR2DL2/HLA-C1C1 pairing may be a risk factor for SARS-CoV-2 infection [36]. The outcomes of their study showed that inhibitory KIRs through their inhibitory signals lead to impaired antiviral function of NK cells. Consequently, the inability of infected cells to effectively eliminate the virus led of more severe clinical symptoms.

A comparison of AA genotype carriers in the two groups of patients and controls in terms of KIR2DS4 variants revealed that the frequency of the del/del genotype was significantly higher in severely ill COVID-19 patients. This result may be explained by the fact that the lack of activating signals for NK in AA genotype carriers homozygous for the KIR2DS4 del variant [13]. The results of this study are consistent with previous observational studies. Two studies on COVID-19 patients have shown that the KIR2DS4 gene is associated with the risk of severe COVID-19, although KIR2DS4 variants were not identified [20, 37]. Interestingly, the frequencies of genotype BB and haplotype B in the mild disease group were significantly higher than in the severe disease group. Haplotype A was more frequent in the severe than the mild disease group, suggesting that people carrying haplotype A and the del/del 2DS4 variant may be at a high risk for COVID-19 severity. In contrast, haplotype B and genotype BB, due to having a greater number of activating genes, could lead to an effective NK cell function, especially at the beginning of the response to the virus. Other interesting findings indicated that all human populations have both group A and B haplotypes, but their distribution varies considerably among different populations. In Africans and Caucasians, haplotypes A and B are equally distributed. Conversely, in Northeast Asians (Chinese, Japanese, and Korean) haplotype A is common, while *genotypes* AB or BB are common in Native Americans, Australians, and Indians [23].

The results of this study are in line with a previous study showing that haplotype A and genotype AA are related to the risk of the severe disease form [20]. Based on the above findings, the BB genotype could be a protective factor for COVID-19, because it is linked to a higher activity of NK cells against viral infections. According to inherited patterns and the numbers of activating and inhibitory KIRs genes, the haplotypes are different in people. The numbers of aKIRs and iKIRs were measured to assess the difference between the numbers of activating or inhibitory KIR genes in the studied groups. The data shows a higher number of aKIR in individuals with mild vs. severe disease (mild group: aKIR = 3, severe disease group: aKIR = 1). Interestingly, the proportion of iKIR > aKIR was higher in the severe disease groups than in the mild disease group. These results agree with the findings of Zaia et al., indicating that recipients of hematopoietic cell transplants from donors with a higher number of aKIR can control early Cytomegalovirus infection and are less likely to be re-infected [38]. However, these results differ from the study by Aranda-Romo et al., which showed that patients with severe influenza infection had ≥ 3 KIR-activating genes; this number was significantly higher than healthy people [34]. The conflicting results may be due to the nature of infectious agent or different number of subjects in the studies. In conclusion, our results confirm previous findings and bring forward additional evidence to suggest that carrying a certain KIR gene content (KIR2DL3,2DS4del and haplotype A) is associated with COVID-19 disease severity. In addition, it can be assumed that inheriting the activating KIR gene set (KIR2DS1, 2DS2, B haplotype, and BB genotype) has a potentially protective effect against severe COVID-19 symptoms. A drawback of our study is that we could not evaluate the HLA types in our patients. However, any subjects with comorbidity were excluded from this study.

## Conclusion

This study showed that the frequency of activating KIRs were higher in patients with mild vs. severe COVID-19. These KIR genes are related to a higher activity of NK cells and may contribute to why patients do not develop severe COVID-19. While these findings align with existing literature, it is important to note, the impact of KIR genes on COVID-19 susceptibility and severity is still an area of ongoing research. More studies are needed to fully understand the relationship between the KIR genetic variations and disease outcomes. In addition, other factors such as age, underlying diseases and various environmental factors may also play a significant role in determining an individual’s susceptibility to severe COVID-19 and should be factored into these studies.

### Electronic supplementary material

Below is the link to the electronic supplementary material.

Supplementary Material 1

Supplementary Material 2

## Data Availability

The datasets generated and/or analyzed during the current study are not publicly available due to privacy and ethical concerns but are available from the corresponding author on reasonable request.

## Abbreviations

KIR: Killer cell Immunoglobulin-like Receptor
COVID-19: Coronavirus disease 2019
WHO: World Health Organization
NK cell: Natural Killer cell
MHC-I: Major Histocompatibility Complex type I
Tel: Telomeric
Cen: Centromeric
SSP-PCR: Polymerase chain reaction-sequence specific
