# Supplementary material for: The effects of killer cell immunoglobulin-like receptor (KIR) genes on susceptibility to severe COVID-19 in the Iranian population
Source: BMC Immunol. 2024 Jun 28;25:38. doi: 10.1186/s12865-024-00631-1 (PMC11212229; doi:10.1186/s12865-024-00631-1)
Supplement: Supplementary file 1 — Supplementary Material 1 [file 12865_2024_631_MOESM1_ESM.docx]

| **Supplementary Table 1:** KIR profile in severe and mild group patients. Gray and white cells indicate the presence and absence of related genes, respectively. | | | | | | | | | | | | | | | | | | | | |
| --- | --- | --- | --- | --- | --- | --- | --- | --- | --- | --- | --- | --- | --- | --- | --- | --- | --- | --- | --- | --- |
| **Genotype** | | | **A haplotype associated** | | | | **B haplotype associated** | | | | | | | **Pseudogenes** | | **Framework** | | | **Frequency** | |
| **Genotype_ID** | **Haplogroup** | **Bx subtype** | ***3DL1*** | ***2DL1*** | ***2DL3*** | ***2DS4*** | ***2DL2*** | ***2DL5*** | ***3DS1*** | ***2DS1*** | ***2DS2*** | ***2DS3*** | ***2DS5*** | ***2DP1*** | ***3DP1*** | ***2DL4*** | ***3DL2*** | ***3DL3*** | **sever(n=198)** | **mild (n=196)** |
| 1 | AA | ̶ |  |  |  |  |  |  |  |  |  |  |  |  |  |  |  |  | 33 | 25 |
| 2 | AB | CxT4 |  |  |  |  |  |  |  |  |  |  |  |  |  |  |  |  | 3 | 2 |
| 3 | AB | CxT4 |  |  |  |  |  |  |  |  |  |  |  |  |  |  |  |  | 2 | 3 |
| 4 | AB | CxTx |  |  |  |  |  |  |  |  |  |  |  |  |  |  |  |  | 7 | 8 |
| 5 | AB | C4Tx |  |  |  |  |  |  |  |  |  |  |  |  |  |  |  |  | 11 | 13 |
| 6 | AB | C4T4 |  |  |  |  |  |  |  |  |  |  |  |  |  |  |  |  | 1 | 7 |
| 7 | AB | C4Tx |  |  |  |  |  |  |  |  |  |  |  |  |  |  |  |  | 5 | 4 |
| 8 | AB | CxTx |  |  |  |  |  |  |  |  |  |  |  |  |  |  |  |  | 2 | 1 |
| 9 | AB | CxTx |  |  |  |  |  |  |  |  |  |  |  |  |  |  |  |  | 0 | 3 |
| 10 | AB | CxTx |  |  |  |  |  |  |  |  |  |  |  |  |  |  |  |  | 3 | 1 |
| 11 | AB | C4Tx |  |  |  |  |  |  |  |  |  |  |  |  |  |  |  |  | 1 | 6 |
| 12 | AB | CxT4 |  |  |  |  |  |  |  |  |  |  |  |  |  |  |  |  | 1 | 3 |
| 13 | AB | C4Tx |  |  |  |  |  |  |  |  |  |  |  |  |  |  |  |  | 2 | 1 |
| 14 | AB | CxTx |  |  |  |  |  |  |  |  |  |  |  |  |  |  |  |  | 2 | 4 |
| 15 | AB | CxTx |  |  |  |  |  |  |  |  |  |  |  |  |  |  |  |  | 0 | 2 |
| 16 | AB | CxTx |  |  |  |  |  |  |  |  |  |  |  |  |  |  |  |  | 2 | 0 |
| 18 | AB | CxT4 |  |  |  |  |  |  |  |  |  |  |  |  |  |  |  |  | 4 | 0 |
| 19 | AB | CxTx |  |  |  |  |  |  |  |  |  |  |  |  |  |  |  |  | 9 | 2 |
| 21 | AB | CxTx |  |  |  |  |  |  |  |  |  |  |  |  |  |  |  |  | 0 | 3 |
| 22 | AB | C4Tx |  |  |  |  |  |  |  |  |  |  |  |  |  |  |  |  | 5 | 0 |
| 23 | AB | CxTx |  |  |  |  |  |  |  |  |  |  |  |  |  |  |  |  | 0 | 4 |
| 24 | AB | CxTx |  |  |  |  |  |  |  |  |  |  |  |  |  |  |  |  | 2 | 0 |
| 25 | AB | C4Tx |  |  |  |  |  |  |  |  |  |  |  |  |  |  |  |  | 6 | 2 |
| 29 | AB | CxTx |  |  |  |  |  |  |  |  |  |  |  |  |  |  |  |  | 1 | 3 |
| 31 | AB | CxTx |  |  |  |  |  |  |  |  |  |  |  |  |  |  |  |  | 10 | 3 |
| 33 | AB | CxTx |  |  |  |  |  |  |  |  |  |  |  |  |  |  |  |  | 5 | 3 |
| 36 | AB | CxTx |  |  |  |  |  |  |  |  |  |  |  |  |  |  |  |  | 4 | 2 |
| 38 | AB | CxTx |  |  |  |  |  |  |  |  |  |  |  |  |  |  |  |  | 2 | 1 |
| 41 | AB | CxTx |  |  |  |  |  |  |  |  |  |  |  |  |  |  |  |  | 1 | 1 |
| 44 | AB | CxTx |  |  |  |  |  |  |  |  |  |  |  |  |  |  |  |  | 1 | 1 |
| 50 | AB | CxTx |  |  |  |  |  |  |  |  |  |  |  |  |  |  |  |  | 1 | 2 |
| 51 | AB | CxTx |  |  |  |  |  |  |  |  |  |  |  |  |  |  |  |  | 3 | 0 |
| 57 | AB | CxTx |  |  |  |  |  |  |  |  |  |  |  |  |  |  |  |  | 2 | 0 |
| 62 | AB | CxTx |  |  |  |  |  |  |  |  |  |  |  |  |  |  |  |  | 6 | 2 |
| 63 | AB | CxTx |  |  |  |  |  |  |  |  |  |  |  |  |  |  |  |  | 2 | 1 |
| 64 | AB | CxTx |  |  |  |  |  |  |  |  |  |  |  |  |  |  |  |  | 4 | 0 |
| 70 | BB | C4T4 |  |  |  |  |  |  |  |  |  |  |  |  |  |  |  |  | 1 | 1 |
| 71 | BB | C4Tx |  |  |  |  |  |  |  |  |  |  |  |  |  |  |  |  | 2 | 8 |
| 78 | BB | CxT4 |  |  |  |  |  |  |  |  |  |  |  |  |  |  |  |  | 0 | 2 |
| 86 | BB | CxT4 |  |  |  |  |  |  |  |  |  |  |  |  |  |  |  |  | 1 | 1 |
| 87 | BB | C4T4 |  |  |  |  |  |  |  |  |  |  |  |  |  |  |  |  | 1 | 2 |
| 90 | BB | C4Tx |  |  |  |  |  |  |  |  |  |  |  |  |  |  |  |  | 3 | 4 |
| 91 | BB | C4Tx |  |  |  |  |  |  |  |  |  |  |  |  |  |  |  |  | 0 | 2 |
| 92 | AB | CxTx |  |  |  |  |  |  |  |  |  |  |  |  |  |  |  |  | 0 | 2 |
| 110 | BB | C4Tx |  |  |  |  |  |  |  |  |  |  |  |  |  |  |  |  | 1 | 0 |
| 113 | BB | C4Tx |  |  |  |  |  |  |  |  |  |  |  |  |  |  |  |  | 0 | 2 |
| 151 | BB | C4Tx |  |  |  |  |  |  |  |  |  |  |  |  |  |  |  |  | 1 | 2 |
| 171 | BB | CxTx |  |  |  |  |  |  |  |  |  |  |  |  |  |  |  |  | 1 | 1 |
| 176 | BB | CxTx |  |  |  |  |  |  |  |  |  |  |  |  |  |  |  |  | 2 | 0 |
| 188 | AB | CxTx |  |  |  |  |  |  |  |  |  |  |  |  |  |  |  |  | 5 | 0 |
| 191 | AB | CxTx |  |  |  |  |  |  |  |  |  |  |  |  |  |  |  |  | 0 | 3 |
| 192 | AB | CxTx |  |  |  |  |  |  |  |  |  |  |  |  |  |  |  |  | 0 | 2 |
| 202 | AB | CxTx |  |  |  |  |  |  |  |  |  |  |  |  |  |  |  |  | 1 | 2 |
| 205 | AB | CxTx |  |  |  |  |  |  |  |  |  |  |  |  |  |  |  |  | 0 | 2 |
| 228 | BB | CxTx |  |  |  |  |  |  |  |  |  |  |  |  |  |  |  |  | 1 | 1 |
| 233 | BB | CxTx |  |  |  |  |  |  |  |  |  |  |  |  |  |  |  |  | 4 | 1 |
| 260 | AB | CxTx |  |  |  |  |  |  |  |  |  |  |  |  |  |  |  |  | 1 | 3 |
| 266 | AB | CxTx |  |  |  |  |  |  |  |  |  |  |  |  |  |  |  |  | 0 | 2 |
| 269 | AB | CxTx |  |  |  |  |  |  |  |  |  |  |  |  |  |  |  |  | 1 | 1 |
| 275 | AB | CxTx |  |  |  |  |  |  |  |  |  |  |  |  |  |  |  |  | 5 | 1 |
| 280 | BB | CxTx |  |  |  |  |  |  |  |  |  |  |  |  |  |  |  |  | 1 | 1 |
| 319 | AB | CxTx |  |  |  |  |  |  |  |  |  |  |  |  |  |  |  |  | 2 | 0 |
| 336 | AB | CxTx |  |  |  |  |  |  |  |  |  |  |  |  |  |  |  |  | 2 | 0 |
| 381 | AB | CxTx |  |  |  |  |  |  |  |  |  |  |  |  |  |  |  |  | 1 | 2 |
| 382 | AB | C4Tx |  |  |  |  |  |  |  |  |  |  |  |  |  |  |  |  | 2 | 2 |
| 415 | AB | CxTx |  |  |  |  |  |  |  |  |  |  |  |  |  |  |  |  | 0 | 4 |
| 558 | BB | CxTx |  |  |  |  |  |  |  |  |  |  |  |  |  |  |  |  | 0 | 2 |
| 681 | BB | CxTx |  |  |  |  |  |  |  |  |  |  |  |  |  |  |  |  | 0 | 2 |
| 691 | BB | CxTx |  |  |  |  |  |  |  |  |  |  |  |  |  |  |  |  | 1 | 2 |
| **Total** | | | | | | | | | | | | | | | | | | | 184 | 173 |

| Supplementary Table 2: KIR profile in patients and healthy controls. 37 unique genotypes were observed in the studied population. Gray and white cells indicate the presence and absence of related genes, respectively. | | | | | | | | | | | | | | | | | | | | |
| --- | --- | --- | --- | --- | --- | --- | --- | --- | --- | --- | --- | --- | --- | --- | --- | --- | --- | --- | --- | --- |
| **Genotype** | | | **A haplotype associated** | | | | **B haplotype associated** | | | | | | | **Pseudogenes** | | **Framework** | | | **Frequency** | |
| **Genotype_ID** | **Haplogroup** | **Bx subtype** | **3DL1** | **2DL1** | **2DL3** | **2DS4** | **2DL2** | **2DL5** | **3DS1** | **2DS1** | **2DS2** | **2DS3** | **2DS5** | **2DP1** | **3DP1** | **2DL4** | **3DL2** | **3DL3** | **Severe (n=198)** | **Mild (n=196)** |
| 180 | AA | ̶ |  |  |  |  |  |  |  |  |  |  |  |  |  |  |  |  | 1 | 0 |
| 27 | AB | CxTx |  |  |  |  |  |  |  |  |  |  |  |  |  |  |  |  | 0 | 1 |
| 30 | AB | CxTx |  |  |  |  |  |  |  |  |  |  |  |  |  |  |  |  | 1 | 0 |
| 35 | AB | CxTx |  |  |  |  |  |  |  |  |  |  |  |  |  |  |  |  | 0 | 1 |
| 37 | AB | CxTx |  |  |  |  |  |  |  |  |  |  |  |  |  |  |  |  | 0 | 1 |
| 43 | AB | CxTx |  |  |  |  |  |  |  |  |  |  |  |  |  |  |  |  | 1 | 0 |
| 46 | AB | CxTx |  |  |  |  |  |  |  |  |  |  |  |  |  |  |  |  | 1 | 0 |
| 56 | AB | C4T4 |  |  |  |  |  |  |  |  |  |  |  |  |  |  |  |  | 1 | 0 |
| 68 | BB | CxT4 |  |  |  |  |  |  |  |  |  |  |  |  |  |  |  |  | 0 | 1 |
| 93 | BB | C4T4 |  |  |  |  |  |  |  |  |  |  |  |  |  |  |  |  | 1 | 0 |
| 94 | BB | C4Tx |  |  |  |  |  |  |  |  |  |  |  |  |  |  |  |  | 0 | 1 |
| 110 | BB | C4Tx |  |  |  |  |  |  |  |  |  |  |  |  |  |  |  |  | 1 | 0 |
| 163 | BB | C4Tx |  |  |  |  |  |  |  |  |  |  |  |  |  |  |  |  | 0 | 1 |
| 187 | BB | CxTx |  |  |  |  |  |  |  |  |  |  |  |  |  |  |  |  | 0 | 1 |
| 193 | AB | CxTx |  |  |  |  |  |  |  |  |  |  |  |  |  |  |  |  | 1 | 0 |
| 200 | AB | CxTx |  |  |  |  |  |  |  |  |  |  |  |  |  |  |  |  | 1 | 0 |
| 201 | BB | C4Tx |  |  |  |  |  |  |  |  |  |  |  |  |  |  |  |  | 0 | 1 |
| 277 | BB | CxTx |  |  |  |  |  |  |  |  |  |  |  |  |  |  |  |  | 0 | 1 |
| 280 | BB | CxTx |  |  |  |  |  |  |  |  |  |  |  |  |  |  |  |  | 0 | 1 |
| 308 | BB | CxTx |  |  |  |  |  |  |  |  |  |  |  |  |  |  |  |  | 0 | 1 |
| 317 | AB | CxTx |  |  |  |  |  |  |  |  |  |  |  |  |  |  |  |  | 1 | 0 |
| 327 | BB | CxTx |  |  |  |  |  |  |  |  |  |  |  |  |  |  |  |  | 0 | 1 |
| 337 | AB | CxTx |  |  |  |  |  |  |  |  |  |  |  |  |  |  |  |  | 0 | 1 |
| 339 | AB | CxTx |  |  |  |  |  |  |  |  |  |  |  |  |  |  |  |  | 0 | 1 |
| 350 | BB | CxT4 |  |  |  |  |  |  |  |  |  |  |  |  |  |  |  |  | 1 | 0 |
| 370 | AB | CxTx |  |  |  |  |  |  |  |  |  |  |  |  |  |  |  |  | 1 | 0 |
| 384 | AB | CxTx |  |  |  |  |  |  |  |  |  |  |  |  |  |  |  |  | 1 | 0 |
| 386 | AB | CxTx |  |  |  |  |  |  |  |  |  |  |  |  |  |  |  |  | 0 | 1 |
| 401 | BB | C4Tx |  |  |  |  |  |  |  |  |  |  |  |  |  |  |  |  | 0 | 1 |
| 403 | BB | CxTx |  |  |  |  |  |  |  |  |  |  |  |  |  |  |  |  | 0 | 1 |
| 466 | BB | CxT4 |  |  |  |  |  |  |  |  |  |  |  |  |  |  |  |  | 0 | 1 |
| 475 | BB | CxTx |  |  |  |  |  |  |  |  |  |  |  |  |  |  |  |  | 0 | 1 |
| 522 | BB | C4Tx |  |  |  |  |  |  |  |  |  |  |  |  |  |  |  |  | 0 | 1 |
| 566 | AB | CxTx |  |  |  |  |  |  |  |  |  |  |  |  |  |  |  |  | 0 | 1 |
| 567 | AB | CxTx |  |  |  |  |  |  |  |  |  |  |  |  |  |  |  |  | 0 | 1 |
| 578 | BB | CxTx |  |  |  |  |  |  |  |  |  |  |  |  |  |  |  |  | 1 | 0 |
| 585 | AB | CxTx |  |  |  |  |  |  |  |  |  |  |  |  |  |  |  |  | 0 | 1 |
| Total | | | | | | | | | | | | | | | | | | | 14 | 23 |
