# Supplementary material for: The effects of killer cell immunoglobulin-like receptor (KIR) genes on susceptibility to severe COVID-19 in the Iranian population
Source: BMC Immunol. 2024 Jun 28;25:38. doi: 10.1186/s12865-024-00631-1 (PMC11212229; doi:10.1186/s12865-024-00631-1)
Supplement: Supplementary file 2 — Supplementary Material 2 [file 12865_2024_631_MOESM2_ESM.docx]

| **Oligonucleotide primers used for SSP KIR genotyping** | | | | |
| --- | --- | --- | --- | --- |
| **Set** | **KIR** | **Forward primer sequence**  **(5’–3’)** | **Reverse primer sequence (5’–3’)** | **PCR product size** |
| **1** | *Internal control* | gaggtaactgtgctcacgaacagc | ggtccataccccagtgcttgagaag | 283 |
| **2** | *KIR2DL1* | gttggtcagatgtcatgtttgaa | cctgccaggtcttgcg | 142 |
| **3** | *KIR2DL2* | aaaccttctctctcagccca | gccctgcagagaacctaca | 142 |
| **4** | *KIR2DL3* | agaccctcaggaggtga | caggagacaactttggatca | 156 |
| **5** | *KIR3DL1* | ccatyggtcccatgatgct  tccatcggtcccatgatgtt | ccacgatgtccagggga | 108  109 |
| **6** | *KIR2DL5* | atctatccagggaggggag | catagggtgagtcatggag | 147 |
| **7** | *KIR2DS1* | tctccatcagtcgcatgag  tctccatcagtcgcatgaa | ggtcactgggagctgac | 96  96 |
| **8** | *KIR2DS2* | tgcacagagaggggaagta | ccctgcaaggtcttgca | 110 |
| **9** | *KIR2DS3* | cttgtcctgcagctcct | gcatctgtaggttcctcct | 158 |
| **10** | *KIR2DS5* | agagaggggacgtttaacc | ctgatagggggagtgagt | 147 |
| **11** | *KIR3DS1* | catcggttccatgatgcg  catcagttccatgatgcg | ccacgatgtccagggga | 107  107 |
| **12** | *KIR2DS4* | cggttcaggcaggagagaat | gtttgaccactcgtagggagc | 199  221 |
